# Supplementary material for: The Footprint of Type 1 Diabetes on Red Blood Cells: A Metabolomic and Lipidomic Study
Source: J Clin Med. 2023 Jan 10;12(2):556. doi: 10.3390/jcm12020556 (PMC9862852; doi:10.3390/jcm12020556)
Supplement: Supplementary file 1 [file jcm-12-00556-s001.zip › SupportingFigureS1.pdf]

A

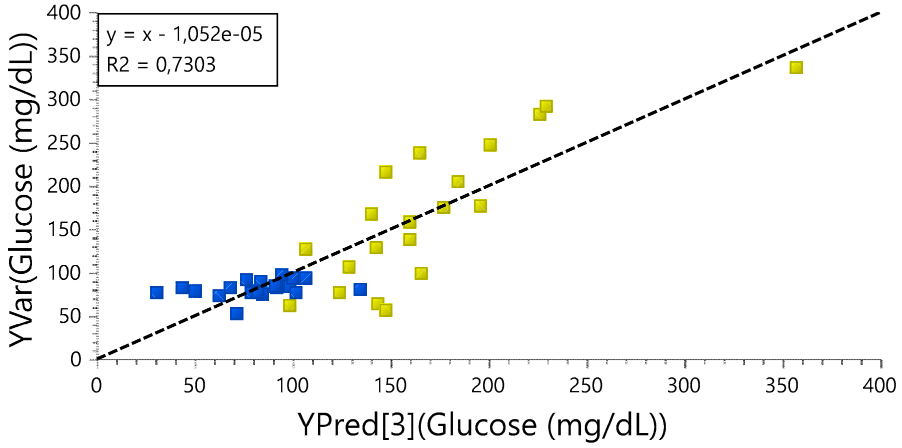

B

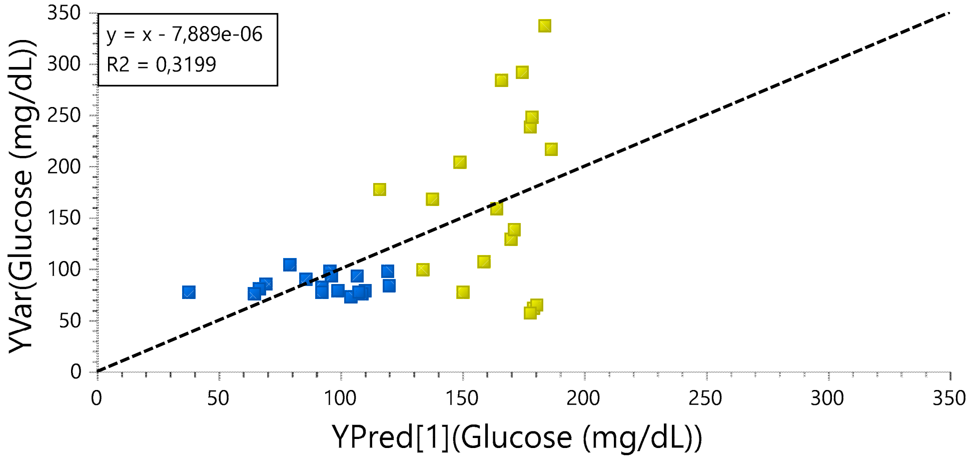

Figure S1. A)PLS correlation of the metabolomics profile with glucose levels in blood. X-axis represents the predicted glucose values, y-axis the experimental glucose values. Data were univariate scaled. Model parameters: R2Y(cum) = 0.73, Q2(cum) = 0.49, CV-ANOVA = 0.0009, permutation validation: R2=(0.0,0.479), Q2=(0.0,-0.304). B)PLS correlation of the lipidomics profile with glucose levels in blood. X-axis represents the predicted glucose values, y-axis the experimental glucose values. Data were univariate scaled. Model parameters: R2Y(cum) = 0.32, Q2(cum) = 0.22, CV-ANOVA = 0.016, permutation validation: R2=(0.0,0.0616), Q2=(0.0,-0.0773).
