# Supplementary material for: The Footprint of Type 1 Diabetes on Red Blood Cells: A Metabolomic and Lipidomic Study
Source: J Clin Med. 2023 Jan 10;12(2):556. doi: 10.3390/jcm12020556 (PMC9862852; doi:10.3390/jcm12020556)
Supplement: Supplementary file 1 [file jcm-12-00556-s001.zip › SupportingTablesS3andS4.pdf]

Table S3. List of metabolites with VIP values >1 in the PLS correlation of the metabolomics profile with glucose levels in blood.

Metabolite	Right limit (ppm)	Left limit (ppm)	VIP	loading (p)
Malonate	3,12	3,128	1.968	0.06
**Phosphocreatine**	3,045	3,052	1.743	0.09
**Phosphocholine**	32,204	32,266	1.727	0.06
Proline	41,231	4,128	1.607	0.04
**3-methyladipate**	0,86	0,911	1.551	-0.13
Choline	3,204	3,217	1.453	0.05
**Propyleneglycol**	11,379	11,565	1.381	-0.09
Glycine	3,553	3,57	1.309	0.09
**2-hydroxyisovalerate**	0,9119	0,9215	1.273	-0.12
Adipate	15,327	15,693	1.215	-0.12
**Formate**	8,454	8,469	1.183	-0.08
**3-methyl-2-oxovalerate**	11,126	11,179	1.148	-0.12
Ornithine	3,054	3,079	1.143	0.03
Proline	4,133	4,155	1.136	0.03
Lysine	15,029	1,526	1.129	-0.12
Betaine	3,264	3,272	1.121	0.05
NADH	8,472	8,489	1.116	-0.03
**Leucine**	0,951	0,9808	1.105	-0.08
Lysine	1,526	1,532	1.104	-0.12
**3-methyl-2-oxovalerate**	11,001	11,069	1.100	-0.12
Phenylalanine	7,362	7,402	1.093	-0.12
AMP	8,597	8,617	1.058	-0.06
**Isoleucine**	1,007	1,027	1.050	-0.11
**Acetate**	1,915	1,925	1.042	-0.06
**Lactate**	41,041	4,123	1.042	-0.02
**Sarcosine**	2,753	2,76	1.016	-0.10
Lysine	1,871	1,909	1.011	-0.06

Table S4. List of metabolites with VIP values >1 in the PLS correlation of the lipidomics profile with glucose levels in blood.

Metabolite	Right limit (ppm)	Left limit (ppm)	VIP	loading (p)
**Lipid -CH2-**	1,21	1,39	1.58353	-0.41
Phospholipids	5,289	5,182	1.52067	-0.40
**Phosphatidycholine**	3,311	3,449	1.42022	-0.37
**Lipid CH3-**	0,79	0,85	1.36112	0.36
Phosphatidylethanolamine	3,235	3,298	1.03254	0.32
Cholesterol	1,002	1,03	0.10325	0.28
